# Interventions to Enhance Resilience Among Family Caregivers: A Systematic Review

**DOI:** 10.1093/geroni/igaa057.494

**Published:** 2020-12-16

**Authors:** Nai-Ching Chi, Soojeong Han, Ying-Kai Fu, Lynn Nakad, George Demiris

**Affiliations:** 1 University of Iowa, Iowa City, Iowa, United States; 2 University of Washington, Seattle, Washington, United States; 3 University of Pennsylvania, Philadelphia, Pennsylvania, United States

## Abstract

Long-term caregiving can negatively impact caregivers’ physical and psychological well-being and are associated with increased morbidity and mortality among caregivers. A more positive caregiving experience is associated with increased coping skills and resilience. Caregiver-targeted interventions designed to promote caregivers’ resilience may lead to improved caregivers’ well-being, the quality of caregiving, and care recipients’ outcome. However, a comprehensive evaluation of effective interventions that enhance resilience among caregivers is lacking. Therefore, the purpose of this study is to critically evaluate existing training programs to inform clinical practice and future program design. A literature review was conducted to search available articles published before June 2018 in databases including PubMed, CINAHL, PsycINFO, and Scopus. Search strategies used index and keyword methods. The inclusion criteria were peer-reviewed, research studies published in English that evaluated an intervention primarily aimed to improve family caregivers of adult patients’ resilience or had a primary outcome of resilience. Seven studies fit the criteria identified. Six studies used psychosocial therapy and one used pharmacological therapy to promote caregivers’ resilience. Several effective resources were identified, such as counseling, self-help manuals, psychoeducation, cognitive behavioral therapies, and the use of an antidepressant. All studies reported positive improvements in caregivers’ outcomes and six studies reported caregivers had improved resilience after receiving interventions. However, most interventions require health professionals to train patients and caregivers for multiple sessions. Future studies should develop feasible, effective, and less labor-intensive interventions, such as self-help manuals, telehealth interventions, or interventions delivered during routine visits.

